# Longitudinal data collection of Mycobacterium avium subspecies Paratuberculosis infections in dairy herds: the value of precise field data

**DOI:** 10.1186/s13567-015-0187-y

**Published:** 2015-06-19

**Authors:** Ynte H Schukken, Robert H Whitlock, Dave Wolfgang, Yrjo Grohn, Annabelle Beaver, JoAnn VanKessel, Mike Zurakowski, Rebecca Mitchell

**Affiliations:** Department of Population Medicine and Diagnostic Sciences, Cornell University, Ithaca, NY 14853 USA; New Bolton Center, University of Pennsylvania, Kennett Square, PA USA; Department of Veterinary Sciences, Pennsylvania State University, University Park, State College, PA 16801 USA; Agricultural Research Services, USDA, Baltimore, MD USA; GD Animal Health, Deventer, the Netherlands

## Abstract

Longitudinal infection data on Mycobacterium avium subspecies paratuberculosis (MAP) was collected on three dairy farms in Northeastern United States during approximately 10 years. Precise data on animal characteristics and animal location within farm were collected on these farms. Cows were followed over time with regard to MAP status during biannual fecal and serum sampling and quarterly serum sampling. Approximately 13 000 serum samples, 6500 fecal samples and 2000 tissue samples were collected during these years. Prevalence of positive samples was 1.4% for serological samples, 2.2% in fecal samples and 16.7% in tissue samples. Infection dynamics of MAP was studied and resulted in a number of potential changes in our understanding of MAP infection dynamics. First, a high prevalence of MAP infection was observed in these herds due to lifetime follow up of cows, including slaughter. Second, two distinctly different infection patterns were observed, so called non-progressors and progressors. Non-progressors were characterized by intermittent and low shedding of MAP bacteria and a virtual absence of a humoral immune response. Progressors were characterized by continuous and progressive shedding and a clearly detectable and progressive humoral immune response. Strain typing of MAP isolates on the three farms identified on two of three farms a dominant strain type, indicating that some strains are more successful in terms of transmission and infection progression. Continuous high quality longitudinal data collection turned out to be an essential tool in our understanding of pathobiology and epidemiology of MAP infections in dairy herds.

## Introduction

Johne’s disease (JD), or paratuberculosis, is a chronic enteric disease of cattle and other ruminants due to an infection with *Mycobacterium avium* subsp. *paratuberculosis* (MAP) [1,2]. Herd-level MAP infection prevalence has gradually increased in the past decade; in a recent survey, it was found that 68% of US dairy herds have apparently at least one cow that is infected with MAP [3]. This estimate was obtained from a survey published by the USDA’s National Animal Health Monitoring System in 2007 [3]. The economic impact of MAP infections to the dairy industry in the United States varies but the cost to dairy producers was estimated to be more than $200 million per year [4].

The epidemiology of MAP in dairy herds is difficult to study as the infection shows a very slow progression from initial infection to clinical disease [5]. Many infected animals never show clinical signs and, many infected animals are only detected using diagnostic tests a few years after initial infection or are actually never detected [1]. Even more, under commercial farm circumstances, diagnostic testing is infrequent and there is a low diagnostic test sensitivity for animals shedding either intermittently or low levels of MAP [6]. Hence, precise information on the infection status of animals is difficult to obtain. Still, the best data necessary to understand epidemiology and pathobiology of MAP will likely be obtained from animals that are studied intensively during their complete lifetime under field conditions on commercial dairy farms [7]. Particularly if the longitudinal study on-farm is followed by culture of tissues at slaughter with a known predilection of MAP infection [8].

An important issue in our understanding infection dynamics of MAP has been the relative low prevalence of animals that are apparently infected (test positive). In most studies, farm prevalences between 3 – 10% are the dominant category [9-11]. With such a low prevalence, infection fade out would be expected in a large proportion of infected farms [12,13]. In reality very few farms, if any, have been reported that successfully eliminated infected. The combination of low prevalence with infection persistence provides for a MAP conundrum with so far not a reasonable rational explanation [14].

The use of field samples is also complementary to studies in animal models. Field studies, of course, do not control the environment, MAP exposure, host and bacterial genotype. Therefore such field data are often difficult to interpret, as the sources of variation are often not well understood. Through genome-wide association studies using high densities of single nucleotide polymorphism markers, joint analysis across animals and herds have become a reality [15,16]. Hence combining precise longitudinal data on infection status and detailed host genetic and bacterial strain type information may now be combined to evaluate the impact of genetic susceptibility to infectious disease such as MAP. In such studies, the choice of the disease phenotype is essential to identify a relevant genetic susceptibility that may be used for selection based disease control programs [17].

To be able to estimate infection incidence and prevalence, longitudinal data will be essential [18]. This is obvious for incidence of infection as animals susceptible to infection will need to be followed to determine when and whether they become MAP infected. However with infections such as MAP with a very slow progression and a long time delay between infection and the first measurable signs of infection, a single cross-sectional measurement will not provide an accurate estimate of infection prevalence [19]. Recent studies have provided initial evidence that transmission routes of MAP include calf-to-calf transmission [20] and adult-to-adult transmission [5]. These routes have typically not been taken into account in MAP control programs. Quantitative estimates of the importance of these transmission routes would be essential to decide on relevant control procedures.

Here we describe the collection and use of long-term longitudinal data on three commercial dairy herds in the North East of the United States. Data were collected for approximately 10 years on these farms. The objective of the paper is to show the particular value of longitudinal data on slow infections such as MAP. Specifically, we describe the long-term collection of data on commercial dairy farms and we try to answer specific questions using longitudinal data:

1. Is the true prevalence based on longitudinal data different from estimates based on cross-sectional data?
2. Based on strain typing of isolates, are there within a herd over time multiple infection dynamics concurrently, rather than a single infection dynamic?
3. Are new infections occurring throughout life, and not just in young animals?

## Materials and methods

**Figure 1:**
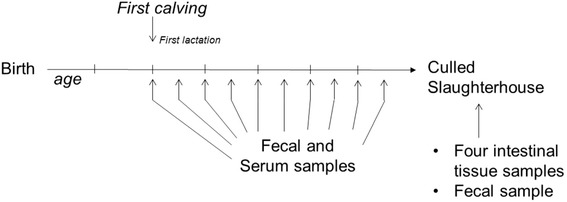
**Sampling scheme in the three RDQMA farms.** All cows in the three farms were sampled at least twice a year from first calving. At each sampling event, both serum and fecal samples were taken. At the time of culling, animals were tagged with special RDQMA ear tags. When these tags were recognized at slaughter, further samples were taken after slaughter. The harvested tissues included two lymph nodes located at the ileocecal junction and two pieces of ileum, one taken from 20 cm proximal to the ileocecal valve and the other taken from very near the ileo-cecal valve. A final fecal sample was also taken at the time of slaughter.

The longitudinal dataset that we will describe here was obtained from three commercial dairy farms in the northeastern United States: farm A in New York State, farm B in Pennsylvania, and farm C in Vermont [5]. All three farms participated in the Regional Dairy Quality Management Alliance (RDQMA) project, which is a multistate research program conducted under a cooperative research agreement between the USDA Agricultural Research Service (ARS) and four Universities, Cornell University, Pennsylvania State University, University of Pennsylvania, and University of Vermont. The project emphasized longitudinal data collection in areas in which infectious diseases of public and animal health concern in dairy herds are endemic. For a more complete description, including information on farms, samplings, and microbial analyses, see Pradhan et al. [5]. Briefly, the milking herds consisted of approximately 330, 105, and 145 cows on farms A, B, and C, respectively. Sampling commenced in February, March, and November 2004 on farms A, B, and C, respectively, and continued for approximately 10 years, for farm A even up until today and still continuing. The project design included biannual collection of individual fecal samples and quarterly serology from all milking and non-lactating cows. Additionally, culled cows were tracked as much as possible from the farm to the slaughterhouse, and at the slaughterhouse four gastrointestinal tissues and a fecal sample were collected with the cooperation of USDA Food Safety and Inspection Service personnel. A summary of the sampling scheme is shown in Figure 1. During the study, the farms remained closed and did not purchase animals. Farm B was constituted from several herds just prior to the start of the study. Farm C included a number of cows from a neighboring dairy for a while due to a barn fire at this neighboring dairy. Farm A was a closed farm for years before the start of the study and remained a closed farm throughout the study. Throughout the study, the farm owners received all results from all tests and were advised with regard to optimal MAP management practices that would result in lower MAP prevalences.

**Figure 2:**
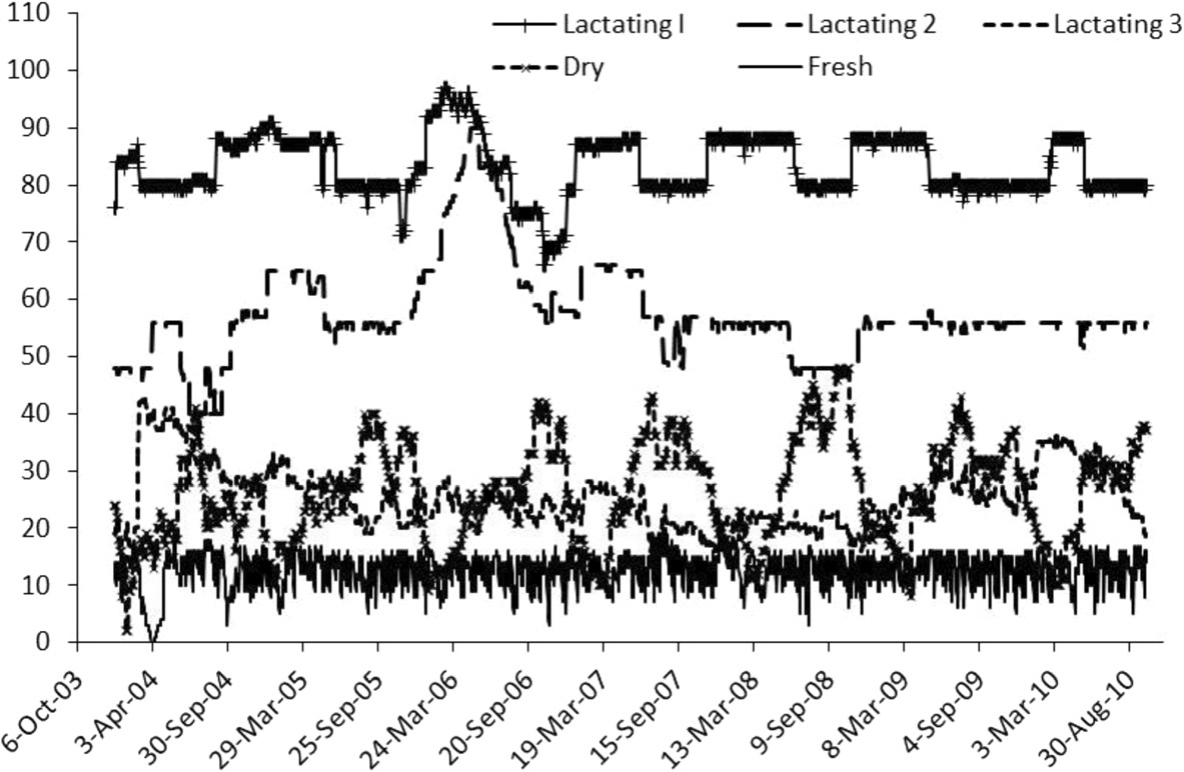
**Cow location per pen from 1/1/2004 and 12/31/2010.** Precise data on cow location within farm was available here for all cows and all pens on one of the farms in the RDQMA study. Shown in this figure are the number of cows present per day in three lactating pens, dry cow pen and fresh pen.

The harvested tissues included two lymph nodes located at the ileocecal junction and two pieces of ileum, one taken from 20 cm proximal to the ileocecal valve and the other taken from very near the ileo-cecal valve. In addition to the sampling of animals, farm environment was sampled in approximately 20 locations on a biannual basis [21]. On each of the farms, demographic data, production data and herd management information was collected. Precise demographic data included birth date, birth location, calving dates, fertility data, animal location data (pen status at any point in time), dry-off dates and eventually culling information and cull dates. This demographic data was collected for all animals present on the farms. Figure 2 shows the number of animals in each pen on the farm over a six year period. For every single day during this time period, animal location on the farm was documented. All diagnostic infection data, strain typing data, herd management, demographic and production data was maintained in a relational database (Microsoft Access).

### ELISA

Upon receipt at the laboratory, blood tubes were centrifuged for 10 min at 900 × *g* and the plasma was separated. Harvested plasma was placed in three 1.5-mL screw capped vials (with a rubber O-ring seal) labeled with farm identification, cow identification, and collection date and stored in a −20 °C freezer or stored in a refrigerator for up to 3 days when they could not be processed immediately. Stored plasma samples were taken from the freezer and allowed to come to room temperature before they were processed for ELISA testing. The sample vial was inverted several times to ensure complete mixing. Plasma samples were evaluated with the ParaCheck (Prionics USA Inc., La Vista, NE; formerly CSL/Biocor) ELISA to monitor serologic status of the cows.

### Microbiology

For fecal samples, 2 g was placed in a 50-mL plastic tube containing 35 mL of water (fecal-water tube). The contents were shaken vigorously and placed on a mechanical shaker for a minimum of 30 min. Following mixing, the sample was allowed to stand at room temperature for 30 min. A 5-mL sample from the top portion of the fecal-water tube was transferred to a second 50-mL plastic centrifuge tube containing 25 mL of 0.9% hexadecylpyridinium chloride in half-strength brain heart infusion broth solution (final concentration of hexadecylpyridinium chloride = 0.75%). Next, tubes were incubated at 35 to 37 °C for 18 to 24 h (decontamination or germination step). Following the germination, tubes were centrifuged for 30 min at 900 × *g*, the supernatant was discarded, and the pellet was resuspended by adding 1 mL of antibiotic brew (1 L of half-strength brain heart infusion broth, 18.5 g/L; amphotericin B, 50 mg/L; nalidixic acid, 100 mg/L; vancomycin, 100 mg/L) followed by vortexing. In the next step (incubation step), the resuspended pellet was incubated overnight or up to a maximum of 3 days at 35 to 37 °C. Following incubation, 4 tubes of Herrold’s egg yolk media (2 in-house and 2 commercial (BD Diagnostics)) were inoculated with 0.2 mL per tube and then incubated in a slanted position at 37 °C. The tubes were read every 2 weeks with the final reading at 16 weeks. Slightly raised white-yellow colonies were evaluated for typical acid fastness and morphological appearance of MAP. Each culture with colony growth was subcultured for mycobactin dependency before reporting the culture positive for MAP.

### MAP Shedding pattern analysis

Animals with at least four data points on bacterial shedding were included in an analysis on MAP shedding patterns. Two types of shedding were recognized, progressors and non-progressors. One group of MAP shedders were indicated as progressors, these animals showed an increase in cfu of MAP over time. In these progressors, no samples were obtained from these animals that were negative for MAP (0 cfu) after previous samples where MAP bacteria were identified. Non-progressors were defined as cows with no increase in cfu of MAP shedding and measurement of absence of shedding in between fecal samples that showed MAP shedding were present in these animals.

### Molecular epidemiology, strain typing methods

The most frequently used method for MAP straintyping is based on sequencing of multilocus short-sequence-repeats (MLSSR). This sequence based method is a highly discriminatory method that has been used for typing M. avium subsp. paratuberculosis isolates and many other bacteria [22]. While only a limited number of cross-sectional studies have used this method, and with a restricted set of isolates, it has been recognized that the use of well-designed longitudinal studies using several herds in multiple states is essential for applying the MLSSR sequencing technique to understand the epidemiology of *M. avium subsp. paratuberculosis* [5]. Six loci were selected due to their highest genetic diversity indices and were identified as the most discriminatory, stable, and informative SSR loci [5]. PCR amplification was carried out with extracted DNA for all isolates using the previously published primers for the six loci [5].

### Estimation of adult exposure as it relates to infection status at slaughter

Using the demographical data for each cow, it was possible to evaluate for every cow in Farm A the daily pen location (see Figure 2 for farm pen data) and therefore the daily configuration of cows in each pen. Combining the pen location data with fecal culture results and molecular typing allowed for strain specific estimation of exposure of each individual cow on a daily basis. For each cow in the herd, we calculated a strain-specific estimate of exposure-days and days without exposure and then regressed this against the cow’s strain-specific infection status at slaughter as the outcome variable (see below).

### Statistical methods

All data were stored in databases and evaluated for missing or unlikely values. Data quality was checked on a continuous basis. Statistical analysis were done in SAS v. 9.3. All data were analyzed using descriptive methods. Data on the risk of infection at slaughter as it relates to exposure to MAP as adult cows was analyzed using logistic regression analysis. The risk of strain-specific MAP infection at slaughter was modeled as a binary variable, and the number of 100 day periods that a cow was in the same pen as a shedder of the same strain was used as the predictor variable. The logistic regression model was then:

$$ \mathrm{Logit}\ \left(\mathrm{MAP}\hbox{-} \mathrm{infecte}{\mathrm{d}}_{\mathrm{Strain}\ \mathrm{i}}\right) = {\upbeta}_0 + {\upbeta}_1*\ \mathrm{Days}\ \mathrm{exposed}\ \mathrm{t}\mathrm{o}\ \mathrm{strai}{\mathrm{n}}_{\mathrm{i}} + \mathrm{error} $$

These analyses on the risk of infection as an adult cow were only done for the dominant strains in farm A.

## Results

Duration of measurements on farms was different between the three farms. Farm A was studied for approximately 10 years and is still being followed. Herd B was in the study for a total of 8 years and herd C was in the study for a total of 7 years. During the years of sampling, a total of approximately 7000 fecal samples, 13 000 serum samples and 1500 tissue samples were collected. These data are summarized in Table 1.

**Table 1:** **Number of samples collected during the course of RDQMA study**

	**Farms**			
	**A**	**B**	**C**	**Total**
**Herd size**	330	105	145	580
**Fecal results**				
Negative	3905	1366	1076	6347
Positive	71	16	56	143
% positive	1.79%	1.16%	4.95%	2.20%
**Tissue results**				
Negative	1274	911	358	1592
Positive	318	76	155	318
% positive	20.0%	7.7%	30.2%	16.7%
**Serological results**				
Negative	7531	2509	2807	12847
Positive	105	17	55	177
% positive	1.38%	0.67%	1.92%	1.36%

### Fecal results

**Figure 3:**
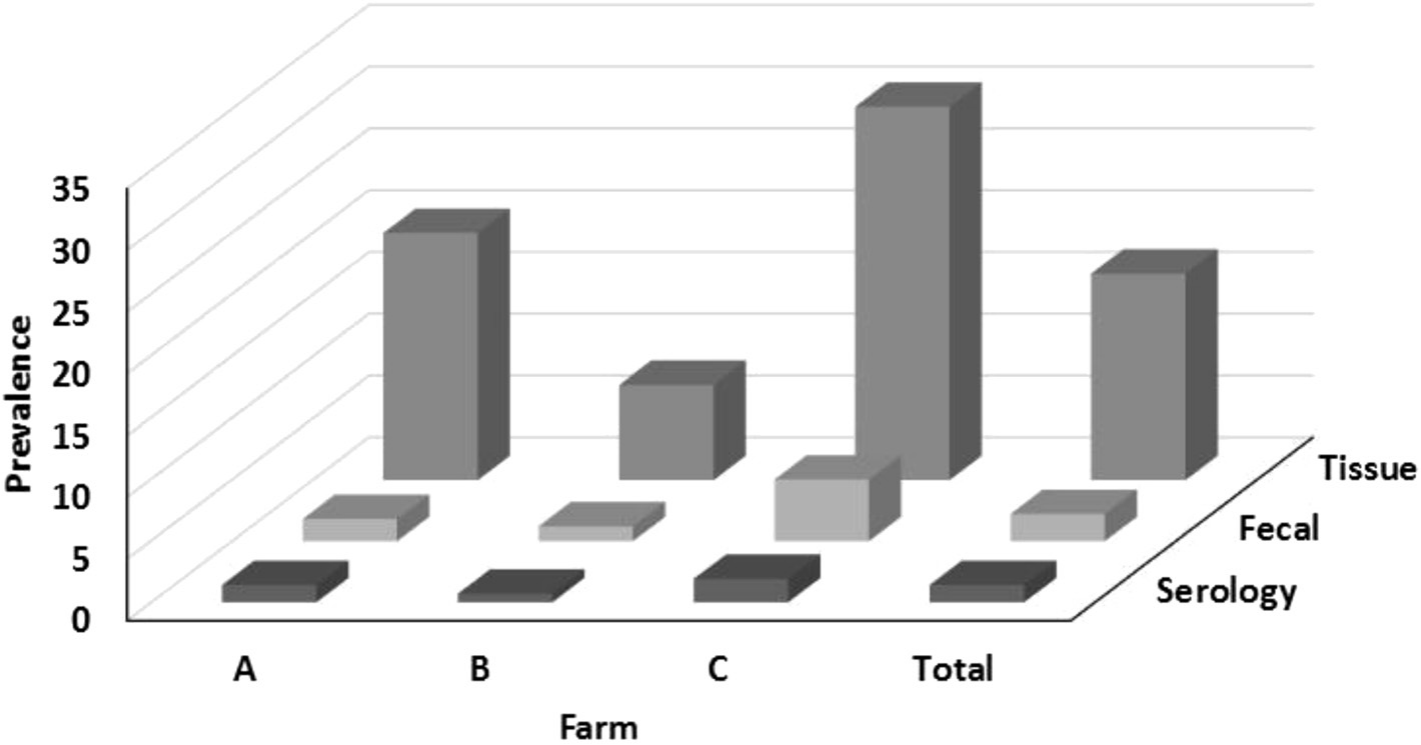
**Serology and culture based MAP prevalence on three RDQMA farms.** Prevalence of MAP in all samples collected throughout a 10 year follow-up study in three dairy farms in New York, Pennsylvania and Vermont. Prevalence in three farms and overall prevalence are shown. Prevalence of MAP in serum samples is measured through ELISA, prevalence in fecal samples is measured using MAP culture methods, prevalence in tissues, collected at slaughter, is measured through culture of four tissues samples.

Prevalence data for fecal results in both fecal samples and in tissues harvested at slaughter are shown in Figure 3 and Table 1. Prevalence in fecal samples ranged between 1.2% and 5.0% of samples with an average of 2.2%. These prevalences are in line with observed prevalence in many surveys [10,18]. However prevalence in tissues was much higher, and ranged between 8% and 30% of all animals with tissue culture results, averaging a prevalence of MAP culture positive of 17% of all cows with tissue culture results.

**Figure 4:**
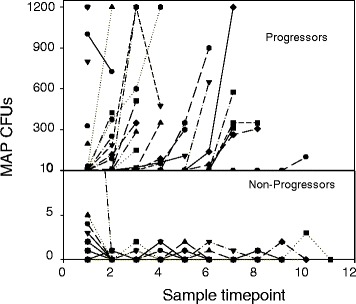
**Longitudinal shedding pattern of cows.** Cows in the top graph are progressing to be a high shedder, while cows in the lower graph are shedding intermittently and low numbers of cfu. Progressors (*n* = 16) were defined as cows with an increasing cfu of MAP shedding over time. Non-progressors (*n* = 42) were defined as cows with no increase in cfu of MAP shedding and measurement of no shedding in between measurements with MAP shedding.

Shedding patterns of 58 individual animals with at least four positive MAP culture results are shown in Figure 4. In Figure 4, animals are split into two groups of shedding patterns. One group of MAP shedders are indicated as progressors (*n* = 16), these animals show an increase in cfu of MAP over time. In these progressors, no samples were obtained from these animals that were negative for MAP (0 cfu) after previous samples where MAP bacteria were identified. Non-progressors (*n* = 42) were defined as cows with no increase in cfu of MAP shedding and measurement of absence of shedding in between fecal samples that showed MAP shedding. These individual shedding patters were used for evaluation of shedding patterns in the companion paper by Mitchell et al. [23]. These longitudinal patterns are valuable to show infection progression, or lack thereof, over time. It appears from these data that animals that eventually become high shedders may be identified early based on their MAP shedding pattern [23].

### Molecular strain typing data

**Figure 5:**
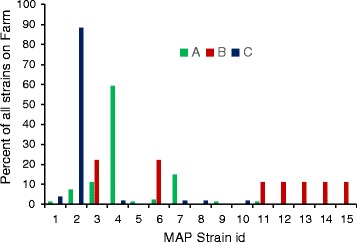
**MAP strain types based on short sequence repeat typing.** In this graph MAP straintyping is based on sequencing of multilocus short-sequence-repeats (MLSSR). Six loci were selected due to their high genetic diversity. PCR amplification was carried out with extracted DNA for all isolates using the previously published primers for the six loci [5]. Strain types were coded using an uninformative coding system, coding sequence types from 1 to 15. Strain diversity per farm is shown in this figure.

In Figure 5, results of MLSSR sequencing technique for discrimination of M. avium subsp. paratuberculosis isolates are shown to describe strain diversity on three farms. It is clear from this figure that multiple infection transmission patterns are present in these herds. Herds A and C show the presence of a dominant strain that is responsible for a large proportion of the observed MAP infections. In contrast, herd B shows the presence of multiple strains at approximately the same frequency. Without having identified the exact transmission pathways, these data appear to indicate that transmission in herds A and C was predominantly due to contagious infection patterns. In herd B this is less obvious, and it may be hypothesized that animals may become MAP infected from multiple sources.

### Serological results

**Figure 6:**
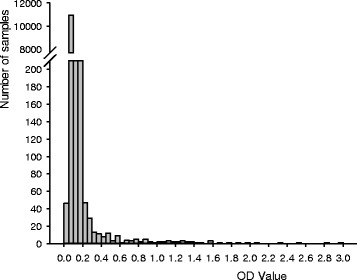
**ELISA MAP results of all samples collected in the study.** Plasma samples were evaluated with the ParaCheck (Prionics USA Inc., La Vista, NE; formerly CSL/Biocor) ELISA. In this figure the optical density value as measured at the end of the ELISA process is shown. Although a cut-off is defined for each batch of samples, the approximate cut-off value for samples to be considered MAP positive is a value greater than 0.20 optical density units. Overall prevalence of positive samples is approximately 1.4%.

**Figure 7:**
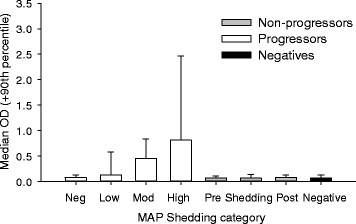
**Distribution of ELISA OD values for progressors and** **non-progressors.** ELISA optical density values are shown for MAP progressors and Non-progressors. Progressors (*n* = 16) were defined as cows with an increasing cfu of MAP shedding over time. Non-progressors (*n* = 42) were defined as cows with no increase in cfu of MAP shedding and measurement of no shedding in between measurements with MAP shedding.

**Figure 8:**
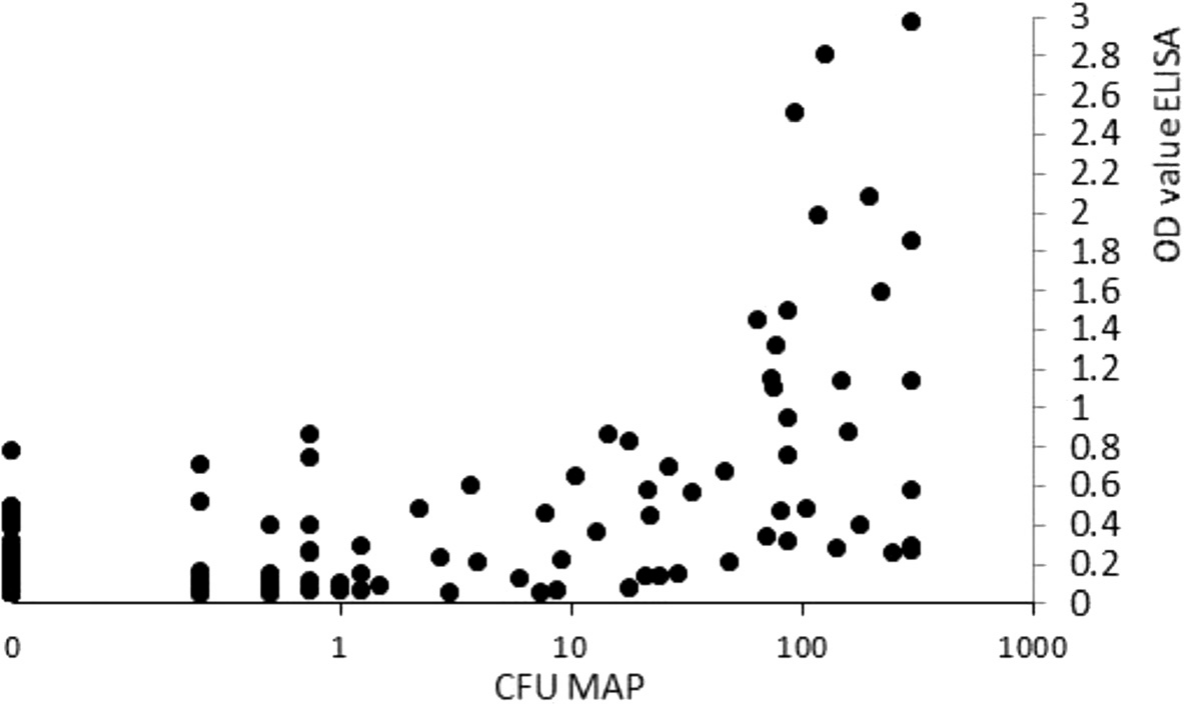
**Relationship between cfu MAP and OD ELISA value.** For this analysis, only samples where Fecal and serum sample were taken on the same day were included in the analysis. CFU values were calculated as the average number of colonies from four culture plates. Values below 1 are possible due to single colonies in only one or more culture plates, but not in all four plates. A total of 10.510 samples were included in the analysis.

Serological results are shown in Figure 6. As expected, the vast majority of animals have low OD values, only a small proportion of animals have OD-values above .2. Although the cut-off for positive results is batch specific, the approximate value for a positive test is an OD value of .2 or greater. Across all farms, only 1.4% of samples were considered serologically positive. Serological results for cows showing infection progression and not showing progression are shown in Figure 7. There was a linear increase in ELISA OD value with increasing CFU in animals that showed an infection progression. Animals that belong to the the non-progressors, had ELISA of values that were low and not significantly different from cows that never shed MAP. In Figure 8, the relationship between cfu of MAP in culture results and ELISA OD value of samples taken at the same time is shown. There was a strong correlation between cfu of MAP and OD ELISA values. Particularly in samples with a cfu close to or more than 100 cfu showed high OD ELISA values. As shown in Figure 7, these higher OD values are virtually only present in animals that show infection progression.

### Adult risk of infection with MAP

**Figure 9:**
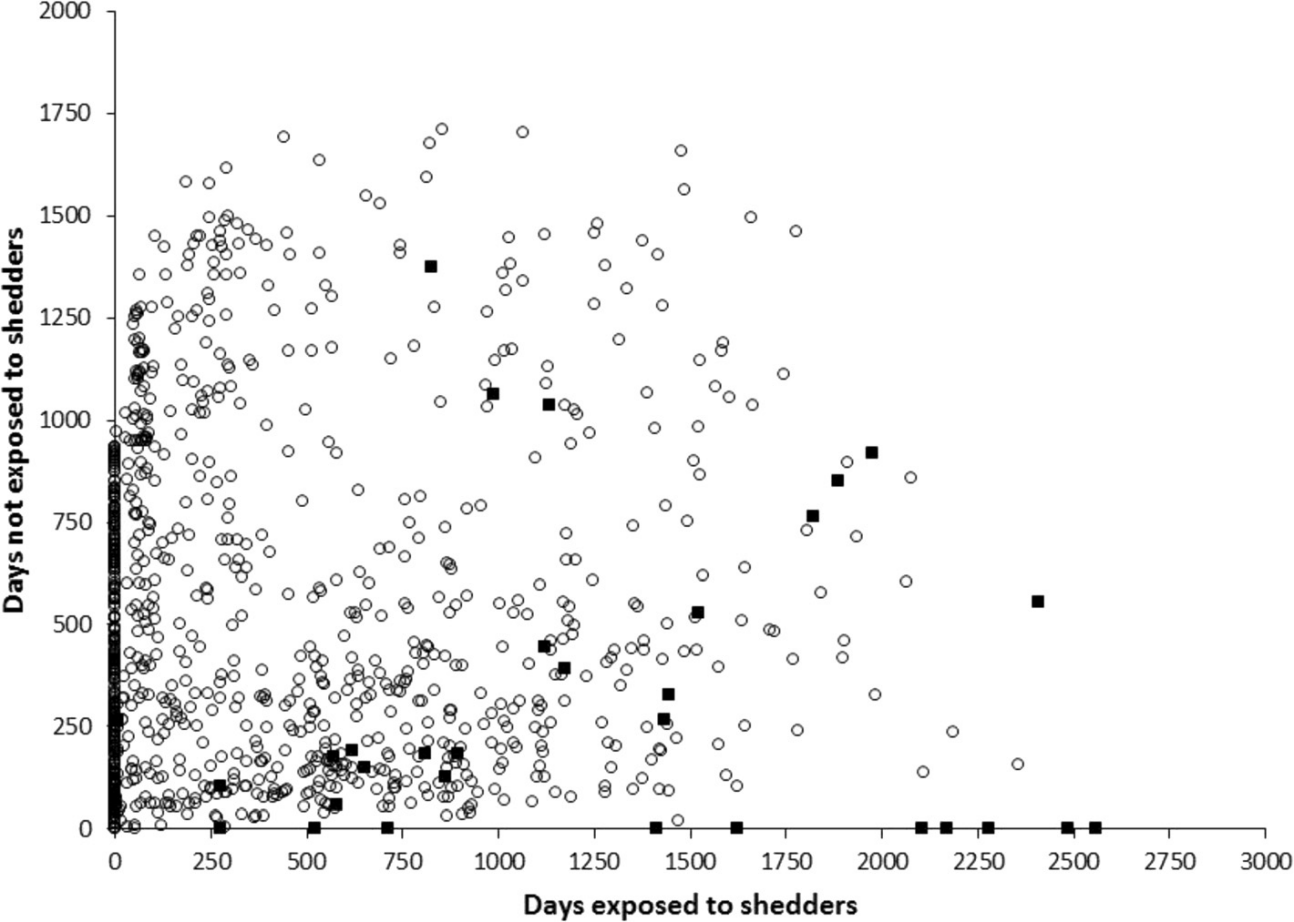
**Lifetime exposure for MAP infected and MAP free cows.** Days exposed and not exposed to MAP shedders for both MAP infected and MAP infection free cows. Where ○ indicates cows that are tissue and fecal culture negative at slaughter, ■ indicates cows that were culture positive in either tissue or fecal at slaughter. Regression analysis showed that per 100 days exposed, cows had an odds ratio of 1.12 (1.06-1.18) to be MAP infected at slaughter compared to MAP unexposed cows.

Data analysis on adult cow physical proximity to shedders and the risk of infection at time of slaughter are shown in Figure 9. Cows that were infected at the time of slaughter with a given strain of MAP were significantly more exposed as adults to cows shedding the same strain of MAP compared to cows that were culture negative for MAP at slaughter. The statistical analysis of these data presented in Table 2 indicated that per 100 days exposed to a heavy shedder, the odds of being infected at slaughter increased significantly with an odds ratio of 1.12 (1.06-1.18). The model fitted the data well, based on the observed AIC value. These results were analyzed for each of the dominant strains on each of the farms (Figures 5 and 9).

**Table 2:** **Logistic regression results of risk of infection at slaughter**

**Parameter**	**Estimate (SE)**	**Chi-square**	***P*** **-value**	**Odds ratio (95% CI)**
Intercept	−5.25 (.48)	118.69	<.0001	
MAP exposure strain_i_	0.11 (.027)	16.56	<.0001	1.12 (1.06 – 1.18)

Cow-specific exposure during their adult life was regressed against risk of infection with the same MAP strain identified at slaughter was.

## Discussion

Particularly with infectious disease with a slow progression such as infections of ruminants with MAP, longitudinal data are essential to understand infection dynamics. In this manuscript, approximately 10 years of longitudinal data with multiple measurement per year was available to understand MAP infection dynamics. Previous reports on MAP infection dynamics assume infection transmission routes through in-utero infection [24], and infection in early life [25]. Typically an exponential decay in the risk of MAP infection with age is assumed, with a zero risk of infection after the first year of life [2,14,19]. Based on the longitudinal data presented in this manuscript, it has been possible to rethink some of the infection transmission routes of MAP in endemically infected herds.

First, the concept of very low prevalence in endemically infected herds was again dismissed based on the observed data. As with many other studies [10,26], the actual number of cows with positive diagnostic tests at any given point in time is low, in our data anywhere from 1% to approximately 5%. If these prevalence data would reflect the true prevalence of MAP infection, it might be expected that many herds just by chance would be able to eliminate MAP infections from the herd [12]. However, the reality is that such herds that eliminated MAP do not exist in large numbers. Unique in the RDQMA data is the longitudinal follow up where a number of animals were followed all the way into the slaughterhouse. It turned out that these data on tissue MAP infection status showed a much higher prevalence, where an overall prevalence of approximately 20% was observed. These results that show high MAP prevalence are in line with slaughterhouse studies of Wells et al. [27] and Vazquez et al. [26]. To match the fecal and serum prevalence data with the tissue culture results, it is necessary that many animals are actually infected, but that only a relatively small portion of these animals are showing fecal shedding or a sero-response. Or, many MAP infected animals show a latent or intermittent shedding stage where no MAP isolated can be found using the current diagnostic methods. Still, this high prevalence is likely necessary to maintain infection in the herd. Further understanding on the importance of latent MAP infections is one of the key research areas to work on in the coming years.

Vazquez et al. [17,26] presented very similar results on MAP prevalence, in a cross section study on 333 randomly selected Holstein-Friesian cows at slaughter, an infection prevalence based on pathology, histology, serology and rtPCR was estimated. Approximately 53% of cows showed histological lesions associated with paratuberculosis, 29% of cows were positive in rtPCR, 14% of cows were positive in tissue culture, while only 6% of cows was positive in serum ELISA. There was a clear increase in immune responsiveness as lesion severity increased [26], very similar to the observed relationship between ELISA results in progressors and non-progressors as observed in our data, although both Vazquez et al. [26] and Wells et al. [27] were both cross-sectional studies. Vazquez et al. [26] introduced the terms latent and patent infections. Here, latent infected animals show focal granulomatous lesions with our without the presence of MAP and little or no ELISA positivity, while patent infected animals show advanced lesions with MAP presence and very high ELISA positivity. Although the Vazquez study was a cross-sectional study, the observed infection dichotomy appears to coincide with progressors and non-progressors in our data.

The distribution where a large proportion of hosts are infected but only very few shed very high numbers of pathogens and suffer clinical signs is also observed with macro-parasite infections [28,29]. This aggregation of shedding patterns is represented by the negative binomial distribution, where a measure of aggregation, together with the mean, is used to describe the distribution of infectious organisms between hosts [28]. This widely observed aggregation in organism burden arises from *heterogeneities* in host populations or in infection pressure. These heterogeneities may be generated by changes in the climate over time or space; genetic differences between hosts [30]; heterogeneity in infection levels, because of host or physiological (age, sex) differences. It is argued [28,29] that the presence of both infectious organisms and the immune response in hosts produces more stable dynamics and lower host population sizes than that observed in the absence of infectious organisms. In evolutionary analyses [30] it can be shown that parasite fecundity is an evolutionarily stable strategy. Phenotypic polymorphisms with respect to immunity in the host species are common and expected in evolutionary stable host strategies. These similarities in infection and host response profiles in macroparasites and microparasites would suggest that endemic infectious diseases such as MAP have an evolutionary background and operate as population tools that result in more stable host populations.

A second observation that may change our thinking on MAP epidemiology is the apparent occurrence of new infections in adults. Based on molecular data, we concluded earlier that such adult infection are indeed likely [5], and now a more quantitative argument where cows exposed to high shedders as an adult, were significantly more likely to be MAP infected at slaughter with the same strain (see also Figure 9). These precise MAP exposure data, combined with previously reported molecular epidemiological data [5] support the occurrence of new MAP infections in adult animals in endemically infected herds. The data available for analysis of exposure information was limited to cows with complete information. These cows differed from all cows particularly in more of these cows being present earlier in the study. This may have resulted in a bias towards cows born earlier in the study. It is not expected though that the biology of MAP infection has changed over the years of the study.

Although adult exposure may lead to a detectable MAP infection, the role of these adult infected animals in infection transmission is still unclear. It may be expected that these adult infections are less likely to show high shedding or severe clinical disease [19]. Still, transmission in utero and transmission from dam to daughter may still be considered realistic possibilities. Certainly the observed relationship between adult exposure and MAP infection creates a much larger window of infection opportunity. Currently known infection routes are then in-utero [24], from dam to daughter [25], from calf-to-calf [20], and young adults and adults from other adults [this manuscript, 5].

These data also emphasize the value of precise infection data, where not only repeated observations per year were used, but also where all MAP isolates where typed using molecular typing methods. These molecular typing methods have recently become available [22] and are valuable to better understand infection dynamics. In reality, on the farms that we observed, multiple infection dynamics occur simultaneously. As a consequence strain specific transmission studies would be necessary. Hence in future observational studies, molecular typing of obtained isolates is essential to understand infection dynamics on farms.

Genetic selection of animals, where animals are preferably selected that are not infected with MAP may need to be re-evaluated [31,32]. When in reality a very large proportion of animals are MAP infected, and show very little immune response or clinical signs, then selection against such an infection state may not be valuable or even feasible. With such high MAP infection prevalence as reported in this study and by the studies of Vazquez et al. [17,26] and Wells et al. [27], elimination of MAP may not be a reasonable objective for genetic selection programs or control programs. Instead, genetic selection against progressors (our data), high shedders or patent infected animals [17] would be more successful in terms of population progression. In a follow-up study to their initial survey, Vazquez et al. [26] evaluated the genetic associations between phenotype and genotype. It may be hypothesized that non-progressors (latent infections) are a preferred response to MAP infection. These non-progressors show limited lesions and a limited humoral response often combined with low or no MAP presence and represent an immune response that prevents animals from developing more severe forms or even bacteriological cure. In contrast, progressors (patent infections) correspond to true failures with a high antibody production and a high MAP load, and detectable and consistent MAP shedding. Genetic selection should then logically be focused on identifying genetic association with the progressor phenotype. As shown by Vazquez et al. [17], a difference in genetic control between MAP infection phenotypes may be present in the population.

Mycobacterial diseases such as Johne’s are extremely difficult to control due to long latent periods, poor diagnostic sensitivity, wildlife and environmental reservoirs of infection, and heterogeneous strain infectiousness. The key to controlling these diseases is an integrated approach to understand the pathways through which pathogen transmission occurs at all levels in an ecosystem: within animals, between individual animals, between livestock and wildlife, and between livestock and the environment [21]. As we are studying agricultural systems, which must be commercially viable, economic decisions play an important role in contact structures, cattle life histories, and control measures. Thus, we must consider the effects of economic determinants on the transmission dynamics of these systems, as well. Traditional a single discipline approach fails to consider the system as a whole. We believe that an ecological approach, simultaneously considering the impact of all aspects of the disease ecosystem, combined with economic analysis will the offer many advantages over past approaches.

Based on observations in our RDQMA data and other studies [10,17,27], MAP control programs may need to be refocused towards control rather than elimination of MAP infections. The more realistic objective would be to reduce or eliminate clinical disease and production losses due to MAP [33,34]. Such programs aimed at control rather than elimination would likely have many similar characteristics compared to current programs. Reduction of exposure in young animals would be a key characteristic, just as identification and culling of known high shedders [12,13], particularly identified progressors. However, additional components would include reduction of exposure in young animals and in adults, again through identification and elimination of progressors. Genetic selection against progressors would be an additional program component as well as identification of strain types in identified MAP isolates. Based on the identified strain types in a given population, more or less aggressive diagnostic and culling programs may be implemented. Clearly, communication of realistic goals to dairy producers in these control programs would be essential. Further research to develop and evaluate such refocused MAP controls will certainly be necessary.

To carry out such research requires not only detailed longitudinal data, such as the RDQMA data described in this paper, but also to develop the methodology to accurately investigate the transmission of pathogens. Recent improvements of typing techniques, which allow differentiation of MAP strains within cattle herds, have allowed researchers to evaluate within-farm distribution of MAP strains [5,22, this manuscript]. Estimation of transmission based on large scale sequencing data has not been applied to MAP modeling efforts, although the results of a recent study using MAP isolates suggested that estimation of transmission pathways using sequencing data is currently feasible and should be used for estimation of transmission pathways of MAP [15]. As bioinformatics tools become faster, easier, and more affordable, their application to disease research has the potential to expand beyond outbreak investigation to elucidating the fundamentals of disease ecology and transmission. However, new methodologies will be needed to tie these emerging tools to existing methods of analysis, including classical epidemiologic models.

The ideal outcome of such research would be to develop a methodology for incorporating whole genome sequencing results in MAP bacterial transmission models [35,36], involving agricultural systems, cattle life-histories, environmental and wildlife reservoirs, and economic decisions. This will allow us to predict the role of each potential source of infection and to recommend control options targeting these sources, expanding the toolbox available to decision makers.

Longitudinal data are essential to understand infection dynamics of slowly progression infections. Infection dynamics of MAP in three US dairy herds was studied for approximately 10 years and these observations resulted in a number of important changes in our understanding of MAP infection dynamics. First, a much higher prevalence of MAP infection was observed in these herds due to lifetime follow up of cows, including slaughter. Approximately 20% of cows turned out to be MAP infected. Second, two distinctly different infection patterns were observed, so called non-progressors and progressors. Non-progressors were characterized by intermittent and low shedding of MAP bacteria and a virtual absence of a humoral immune response. Progressors were characterized by continuous and progressive shedding and a clearly detectable and progressive humoral immune response. Strain typing of MAP isolates on the three farms identified on two of three farms a dominant strain type, indicating that some strains are more successful in terms of transmission and infection progression. Based on these observations, control programs including specific genetic selection may need to be refocused. Continuous high quality longitudinal data collection turned out to be an essential tool in our understanding of pathobiology and epidemiology of MAP infections in dairy herds.

## Abbreviations

MAP: Mycobacterium avium subspecies paratuberculosis
RDQMA: Regional Dairy Quality Management Alliance
